# In-depth characterization of a mouse model of post-traumatic epilepsy for biomarker and drug discovery

**DOI:** 10.1186/s40478-021-01165-y

**Published:** 2021-04-26

**Authors:** Rossella Di Sapia, Federico Moro, Marica Montanarella, Valentina Iori, Edoardo Micotti, Daniele Tolomeo, Kevin K. W. Wang, Annamaria Vezzani, Teresa Ravizza, Elisa R. Zanier

**Affiliations:** 1 Laboratory of Experimental Neurology, Department of Neuroscience, Istituto Di Ricerche Farmacologiche Mario Negri IRCCS, Via Mario Negri 2, 20156 Milan, Italy; 2 Laboratory of Acute Brain Injury and Therapeutic Strategies, Department of Neuroscience, Istituto Di Ricerche Farmacologiche Mario Negri IRCCS, Via Mario Negri 2, 20156 Milan, Italy; 3 Laboratory of Biology of Neurodegenerative Disorders, Department of Neuroscience, Istituto di Ricerche Farmacologiche Mario Negri IRCCS, Via Mario Negri 2, 20156 Milan, Italy; 4 Program for Neurotrauma, Neuroproteomics and Biomarkers Research, Departments of Emergency Medicine, Psychiatry, Neuroscience and Chemistry, University of Florida, Gainesville, FL USA

## Abstract

Post-traumatic epilepsy (PTE) accounts for 5% of all epilepsies and 10–20% of the acquired forms. The latency between traumatic brain injury (TBI) and epilepsy onset in high-risk patients offers a therapeutic window for intervention to prevent or improve the disease course. However, progress towards effective treatments has been hampered by the lack of sensitive prognostic biomarkers of PTE, and of therapeutic targets. There is therefore a pressing clinical need for preclinical PTE models suitable for biomarker discovery and drug testing. We characterized in-depth a model of severe TBI induced by controlled cortical impact evolving into PTE in CD1 adult male mice. To identify sensitive measures predictive of PTE development and severity, TBI mice were longitudinally monitored by video-electrocorticography (ECoG), examined by MRI, and tested for sensorimotor and cognitive deficits and locomotor activity. At the end of the video-ECoG recording mice were killed for brain histological analysis. PTE occurred in 58% of mice with frequent motor seizures (one seizure every other day), as determined up to 5 months post-TBI. The weight loss of PTE mice in 1 week after TBI correlated with the number of spontaneous seizures at 5 months. Moreover, the recovery rate of the sensorimotor deficit detected by the SNAP test before the predicted time of epilepsy onset was significantly lower in PTE mice than in those without epilepsy. Neuroscore, beam walk and cognitive deficit were similar in all TBI mice. The increase in the contusion volume, the volume of forebrain regions contralateral to the lesioned hemisphere and white matter changes over time assessed by MRI were similar in PTE and no-PTE mice. However, brain histology showed a more pronounced neuronal cell loss in the cortex and hippocampus contralateral to the injured hemisphere in PTE than in no-PTE mice. The extensive functional and neuropathological characterization of this TBI model, provides a tool to identify sensitive measures of epilepsy development and severity clinically useful for increasing PTE prediction in high-risk TBI patients. The high PTE incidence and spontaneous seizures frequency in mice provide an ideal model for biomarker discovery and for testing new drugs.

**Supplementary Information:**

The online version contains supplementary material available at 10.1186/s40478-021-01165-y.

## Introduction

Traumatic brain injury (TBI) is a leading cause of mortality and morbidity. While early life-saving treatments have improved patient survival, preventive therapeutic interventions for arresting or mitigating the late neurological sequelae such as post-traumatic epilepsy (PTE) are still urgently required. PTE accounts for 5% of all epilepsies and 10–20% of the acquired forms [1] with approximately 20,000 new PTE patients a year in the US [2]. A population-based study showed that the 30-year cumulative incidence of PTE is 1.2% for mild, 4.2% for moderate, and 16.7% for severe TBI [3]. The latency from TBI to the first unprovoked seizure varies widely with about 50% of PTE cases diagnosed within the first year and up to 80% within the first 2 years [4, 5]. This lag of time from TBI to PTE provides an ideal therapeutic window for interventions to prevent PTE [2].

There are still no anti-epileptogenic or disease-modifying treatments for PTE, and anti-seizure drugs, while preventing early symptomatic seizures after TBI, do not prevent epilepsy [6–9]. The development of preventive interventions has been hampered by the limited knowledge of the mechanisms of TBI-induced epileptogenesis, and by the lack of prognostic biomarkers to identify subjects at risk for PTE, or predict therapeutic drugs’effects. These steps are instrumental for enriching the patient population who might develop PTE, enabling us to design affordable clinical trials. There is therefore an urgent need for the characterisation and validation of robust preclinical PTE models to foster the discovery of both the molecular mechanisms and the biomarkers of the transition from TBI to PTE, and to test anti-epileptogenic interventions [7, 9].

Experimental models of TBI are a valuable tool for mimicking key clinical neuropathological features of PTE patients, including behavioral deficits, progressive neurodegeneration, white matter damage, and neuroinflammation [10–12]. Late occurrence of spontaneous seizures has been reported after fluid percussion injury in rats [13], a model of closed head injury recapitulating rapid acceleration and deceleration of the head in humans resulting in both focal and diffuse axonal injury, or repetitive TBI by weight drop in mice [14] mimicking sport-related injuries. However, PTE incidence reaches 50% in the rat model only after a very long follow-up (12 months) [13], and the daily seizure frequency in the mouse model is low (0.086) [14]. Conversely, contusional TBI evoked by controlled cortical impact (CCI) in mice may induce PTE with a high incidence and frequent seizures in relatively shorter post-injury times (3–5 months), depending on the genetic background and injury severity. In particular, CD1 mice have a higher PTE incidence than C57/BL6 mice [15, 16], from 20% after mild CCI (0.5 mm depth) [15], to 36–40% after moderate (1 mm depth) [15, 17, 18], and up to 50% after severe injury (2 mm depth) [19]. CD1 mice exposed to severe contusional TBI may therefore serve as a model with the highest PTE development, thus facilitating biomarker discovery and drug testing. Notably, retrospective studies showed that patients with contusive/haemorrhagic injury were at high risk of PTE, with a higher prevalence after parieto-temporal lesion [1, 20–22], and PTE incidence increasing with injury severity [3, 23]. However, although the CCI model in CD1 mice looks promising in the study of PTE, it still lacks a full characterization of the effects of TBI on neurological and neuropathological outcomes.

The purpose of this study was to perform an in-depth characterisation of TBI sequelae with special focus on PTE in CD1 mice exposed to severe left parieto-temporal CCI. We did extensive longitudinal electrocorticography (ECoG) monitoring of spontaneous seizures, and assessed neurological functions with a battery of behavioral tests. We also investigated brain structural alterations by MRI and post-mortem histology. Finally, by correlating these measures with spontaneous seizures in mice, we provide evidence for potential biomarkers of PTE and its severity.

## Material and methods

### Animals

Eight-week-old CD1 male mice (~ 25–30 g; Envigo, Italy) were maintained in SPF facilities and housed at constant room temperature (23 °C) and relative humidity (60 ± 5%) with free access to food and water and a fixed 12 h light/dark cycle. Mice were individually housed with environmental enrichment (toilet paper, straw, nesting material) [24].

Procedures involving animals and their care were conducted in conformity with institutional guidelines that are in compliance with national (D.L. n.26, G.U. March 4, 2014) and international guidelines and laws (EEC Council Directive 86/609, OJ L 358, 1, December 12, 1987, Guide for the Care and Use of Laboratory Animals, U.S. National Research Council, 1996; laws of the United States and regulations of the Department of Agriculture), and were reviewed and approved by the intramural ethics committee and by the Animal Care and Use Review Office (ACURO; mice in cohort 2, see below). All animal experiments were designed in accordance with Animal Research Reporting of In Vivo Experiments (ARRIVE) guidelines [25], with a commitment to refinement, reduction, and replacement, and using biostatistics to optimise the number of mice.

### Experimental design

**Fig. 1:**
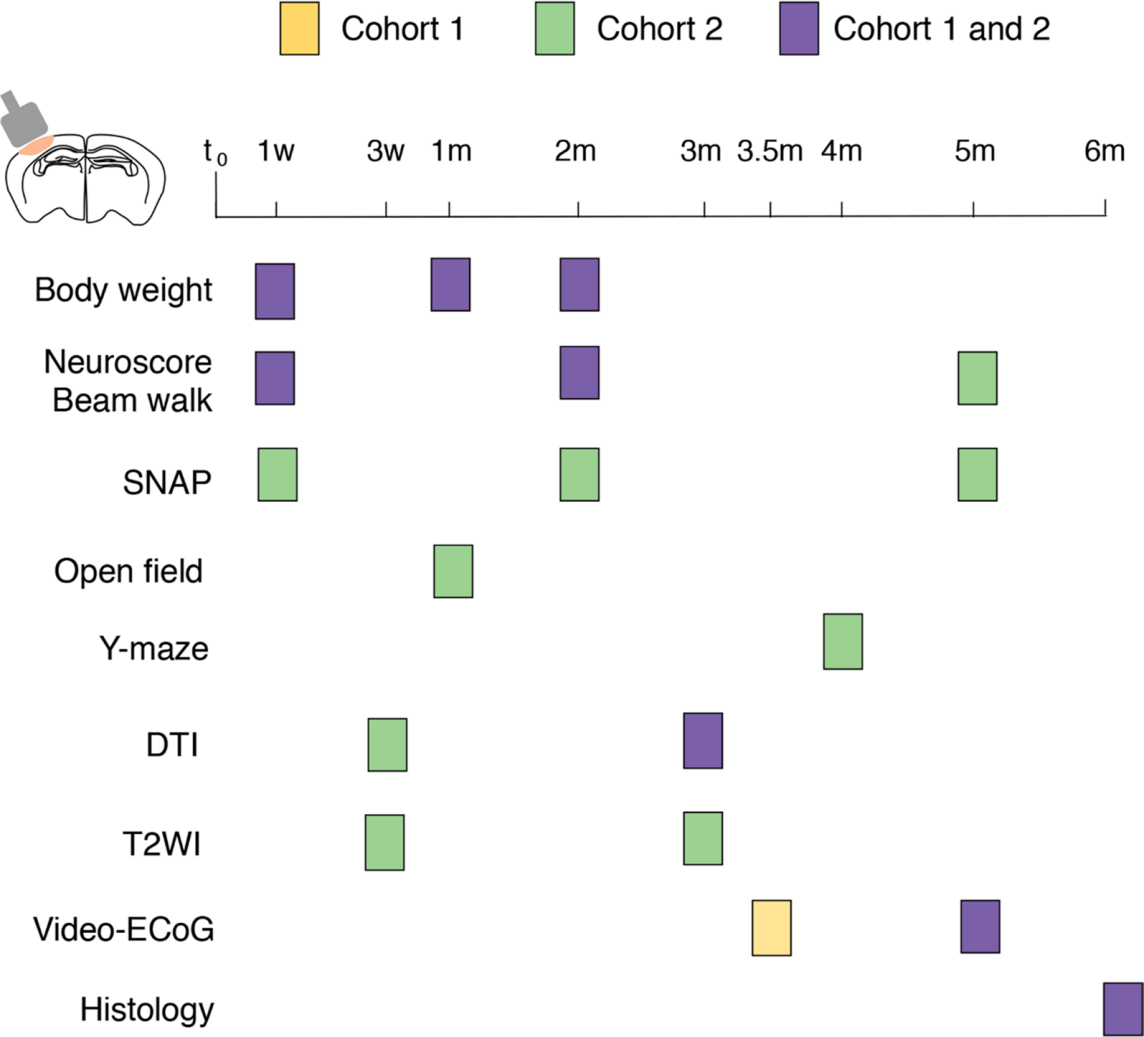
Schematic representation of the experimental design. Two independent cohorts of mice (cohorts 1 and 2) were included in the longitudinal study. TBI was induced by controlled cortical impact (CCI) on the left parieto-temporal cortex in anesthetised mice. The yellow and green boxes indicate measurements in respectively cohort 1 or cohort 2. Purple boxes stand for measures done in both cohorts 1 and 2. t_0_, day of CCI; w, week; m, month; DTI, diffusion tension imaging; ECoG: electrocorticography; SNAP, Simple Neuroassessment of Asymmetric Impairment; T2WI, T2-weighted imaging

Figure 1 shows the timeline of the experiments. We prepared two independent cohorts of TBI and their respective control mice (cohorts 1 and 2). In cohort 1, we ran a longitudinal ECoG monitoring 3 and 5 months after TBI (24/7, 3 weeks each recording session) to measure PTE incidence, and the frequency and duration of spontaneous seizures. We selected these two time points on the basis of previous evidence that they illustrate the early and late (chronic) epilepsy phases in the model, respectively [19]. Sensorimotor deficits and white matter damage were evaluated in the same mice.

In a subsequent cohort 2, we ran a longitudinal study to characterise behavioral (sensorimotor deficits, as well as motor and cognitive tests) and brain structural changes by MRI, with the main aim of identifying biomarkers of PTE development. In this cohort, mice with or without spontaneous seizures were identified at 5 months post-TBI only (chronic phase of epilepsy) since a progression in the incidence of PTE was observed between 3 and 5 months in cohort 1.

Histopathology was evaluated at the end of the experiments in mice taken at random from cohorts 1 and 2.

### Induction of traumatic brain injury

Mice (n = 45) were anesthetized with isoflurane inhalation (2% for induction, 1.5% for maintenance) in an N_2_O/O_2_ (70%/30%) mixture and placed in a stereotaxic frame, then subjected to craniectomy followed by induction of CCI brain injury as previously described [26–29]. Briefly, the injury was induced using a 3 mm diameter rigid impactor driven by a pneumatic piston rigidly mounted at an angle of 20° from the vertical plane and applied perpendicularly to the exposed dura mater, between bregma and lambda, over the left parieto-temporal cortex (antero-posteriority: − 2.5 mm, laterality: left 2.5 mm), at an impactor velocity of 5 m/s and deformation depth 2 mm, resulting in severe injury [19, 30]. The craniectomy was then covered with a cranioplasty and the scalp sutured.

Control sham mice received identical anesthesia, and surgery, but no brain trauma. During all surgical procedures, mice were maintained at a body temperature of 37 °C.

### Behavioral tests

#### Sensorimotor function

Sensorimotor deficits were evaluated by neuroscore, beam walk and Simple Neuroassessment of Asymmetric Impairment (SNAP) tests at specific time points (Fig. 1), as previously described [26–29, 31, 32].

#### Neuroscore

Mice were scored from 4 (normal) to 0 (severely impaired) for each of the following: (1) forelimb function while walking on the grid and flexion response during suspension by the tail; (2) hindlimb function while walking on the grid and extension during suspension by the tail; (3) resistance to lateral right and left pushes (best score 12) [26–29, 31].

#### Beam walking test

Mice were analysed for motor coordination and balance by measuring the number of foot faults of a trained mouse walking twice on an elevated, narrow wooden beam (5-mm wide, 100 cm long). Before each test, mice were trained in three habituation trials. Performance was scored as the sum of the number of foot faults during the two tests (best score 0) [32].

#### SNAP test

Neurological parameters were assessed in eight tests including vision, proprioception, motor strength and posture, as previously described [32]. The result obtained in each of the eight tests was summed to obtain the overall SNAP score. A neurologically intact animal is expected to have a SNAP score of 0.

#### Open field

Mice were placed in the center of a square arena with walls (40 × 40 × 30 cm) with the floor divided into 25 squares (8 × 8 cm). The nine central squares (24 × 24 cm) represent the “central area” and the surrounding border zone the “outer squares”. Mice were tested under dim illumination. Their behaviour was then video-recorded for 5 min (Ethovision XT 5.0, Noldus). The total distance moved (a measure of locomotor activity), and the time spent in the central area (a measure of thigmotaxis and indicative of anxiety-related behavior) were recorded [33].

#### Y maze

The Y maze is a two-trial spatial recognition memory test where performance does not involve learning a rule, but is based on the innate propensity of rodents to explore new environments (i.e. not encountered before). The Y maze apparatus consisted of three arms joined in the middle to form a “Y”. The insides of the arms are identical, providing no intra-maze cues. Visual cues were placed around the perimeter of the maze, and kept constant during the behavioral test. During the acquisition phase (trial 1), one arm of the Y maze was closed with a guillotine door. The position of the closed arm was chosen randomly among the three arms. Each mouse was placed in one of the other two open arms (“starting arm”), and allowed to visit the two accessible arms of the maze for 5 min. At the end of the trial, mice were placed in their home cage. After an inter-trial interval of 1 h, mice were placed in the same “starting arm” as in trial 1, with free access to all three arms for 5 min (trial 2, retrieval phase) [34, 35]. During each trial, the number and the duration of visits to each arm were recorded (Ethovision XT 5.0, Noldus).

### Magnetic resonance imaging analysis

Animals were anesthetized with isoflurane in a mixture of O2 (30%) and N2O (70%). Body temperature was maintained at approximately 37 °C by a warm water-circulated heating cradle, and the respiratory rate was continuously monitored. Experiments were done on a 7 T Bruker Biospec 70/30 Avance III system, equipped with a 12 cm diameter gradient coil (400 mT/m maximum amplitude) (Bruker Biospin, Ettlingen, Germany). Due to a hardware upgrade between experimental sessions, two different coils setup had to be employed. A transmit cylindrical radiofrequency (rf) coil (7.2 cm inner diameter) for transmission and a quadrature receive surface rf (2 × 2 cm) coil array positioned over the animal’s head were used for cohort 1, while a quadrature cryogenic surface coil was used as transmitter and receiver for cohort 2.

In the same imaging session, two subsequent protocols were applied to monitor the structural alterations (2D T2-weighted sequence) and white matter damage (diffusion tensor images, DTI). A 2D T2-weighted SE RARE sequence of the whole brain was done. The images were obtained with a voxel size of 100 × 100 µm (matrix size 150 × 150 and field of view 1.5 × 1.5 cm), slice thickness 0.3 mm, repetition time [TR] = 7500 ms, effective echo time [TE] = 66 ms. MRI images were analysed to measure the volume of selected brain regions using the automatic multiatlas-based segmentation approach embedded in the ANTs software library [36, 37]. The brain regions of interest in the hemisphere contralateral to injury were co-registered on the mouse brain histology atlas of Franklin and Paxinos [38]. Every MRI section was visually inspected, and the brain regions manually corrected, if needed, to take account of the cavity in the TBI group. Measurements were taken using freely available ITK-SNAP software [39].

An echo-planar imaging (EPI) sequence of the whole brain was acquired with an in-plane image. The images were obtained with a voxel size of 125 × 125 µm (matrix size 120 × 120 and field of view 1.5 × 1.5 cm), slice thickness: 0.3 mm, repetition time [TR] = 7000 ms, effective echo time [TE] = 27.5 ms, t and 2 repetitions. Each repetition was co-registered and averaged to increase the signal-to-noise ratio (SNR) after correcting EPI distortions between repetitions. Diffusion, encoding b factors of 800 mm^2^/s is applied along 19 isotropic directions and two B0 unweighted images for each repetition. The diffusion tensor was computed using FSL software. A group mean full tensor template was first created using a population-based DTI atlas construction algorithm that adopts a tensor-based registration procedure embedded in the DTI-TK software library [40]. The average template was resampled to an in-plane resolution of 100 × 100 μm^2^ and a slice thickness of 0.2 mm, and is thinned to obtain a mean skeleton. DTI metrics (fractional anisotropy, FA; axial diffusivity, AD; radial diffusivity, RD; mean diffusivity, MD) were normalized to a mean template with a diffeomorphic transformation, then projected onto the mean skeleton for ROI-based analysis. The SNR within the regions of interest (ROIs) did not differ across experimental groups (sham: 26.03 ± 2.5; PTE: 27.5 ± 3.2; No-SRS: 27.2 ± 2.3), and it was higher than 20, thus allowing to perform reliable quantification of DTI metrics [41]. Reproducibility of the DTI measurements was also checked, and the coefficient of variation was below the 5% critical threshold (range: 2.6–3.8%) [42].

### Detection and quantification of spontaneous seizures

**Fig. 2:**
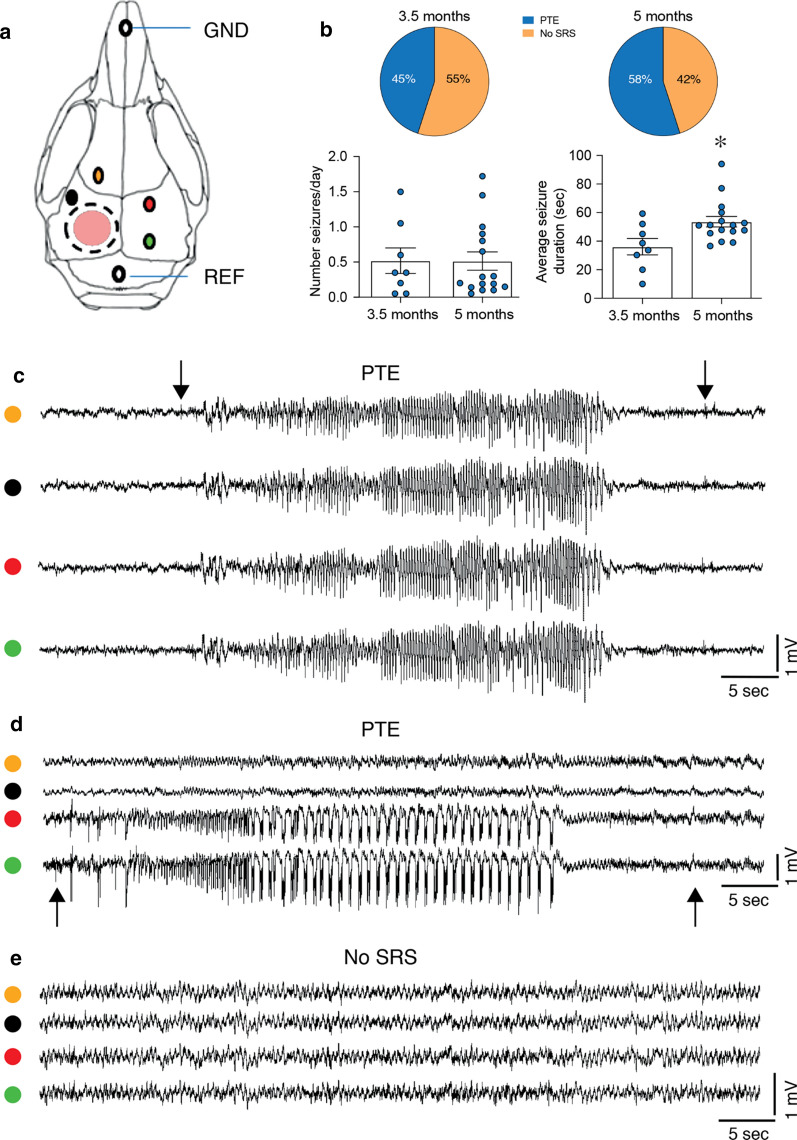
Longitudinal ECoG analysis for monitoring PTE development. **a** Schematic skull reproduction depicting the placement of cortical electrodes used for the ECoG recording (colored dots), ground (GND) and reference (REF) electrodes, the craniectomy (dotted circle), and the CCI injury (pink circle). **b** Upper row: pie charts showing the percentages of mice with PTE (blue) and without spontaneous recurrent seizures (No-SRS; orange) at the two recording periods (TBI mice, 3.5 months: n = 18; 5 months n = 29). Bottom row: bargrams depicting the number of daily SRS and their average duration in PTE mice (3.5 months: n = 8; 5 months: n = 16; one PTE mouse in cohort 1 had SRS at 3.5 months but not at 5 months). SRS were detected by 3-week (24/7) video-ECoG monitoring at each time point. Data are presented as bargrams depicting the mean ± SEM and the single values. *p < 0.05 versus 3.5 months by two tailed unpaired t-test. **c**, **d** Representative ECoG tracings of SRS recorded 5 months post-TBI in two different PTE mice. ECoG seizures may occur bilaterally (**c**), or unilaterally in the contralateral hemisphere (**d**). Black arrows indicate the beginning and end of the seizure events. **e** Depicts a representative ECoG tracing of a No-SRS mouse. The color code at the beginning of the traces identifies the recording electrode (see **a**)

At 3 months (cohort 1) or 5 months post-TBI (cohort 2) (Fig. 1), mice were surgically implanted under general gas anesthesia (1–3% isoflurane in O2) and stereotaxic guidance [43]. Four epidural screw electrodes were implanted in the skull, two ipsilaterally and two contralaterally to the injured hemisphere (Fig. 2a).

The electrodes were positioned from bregma, as follows (Fig. 2a): perilesional cortex (mm: nose bar 0; anteroposterior − 1, lateral left 2.8; black dot); frontal cortex (mm: nose bar 0; anteroposterior + 1.6, lateral left 1.8; yellow dot); parieto-occipital cortex (mm: nose bar 0; anteroposterior − 1 and − 3, lateral right 2.8; green and red dots, respectively). Two additional screw electrodes were positioned over the nasal sinus and the cerebellum, and used as ground and reference electrodes, respectively. Electrodes were connected to a multipin socket and secured to the skull with acrylic cement.

We reckoned the number and duration of spontaneous recurrent seizures (SRS) in electrode-implanted mice by continuous (24/7) video-ECoG monitoring for 3 weeks in each recording period. Seizure frequency was estimated by dividing the total number of seizures by the number of recording days.

Spontaneous seizures consist of ECoG paroxysmal events characterised by high frequency (> 5 Hz, usually 7–10 Hz) and/or multispike complexes and/or high amplitude (700 μV–1.0 mV vs 100–300 μV at baseline) spikes. ECoG activity was recorded using the Twin EEG Recording System (version 4.5.3.23) connected with a Comet AS-40 32/8 Amplifier (sampling rate 400 Hz, high-pass filter 0.3 Hz, low-pass filter 70 Hz, sensitivity 2000 mV/cm; Grass-Telefactor, West Warwick, R.I., USA). ECoG seizures were accompanied by generalized motor convulsions recorded using WFL-II/LED15W infrared video-cameras (Videor Technical, GmbH, Germany) synchronized with the ECoG recording system. Digitized video-ECoG data were processed using the Twin record and review software. ECoG analysis of seizures was done by two independent investigators blinded to the treatment, who reviewed all the ECoG tracings in the electronic files of each mouse. Deviation of ≤ 5% from concordance was considered acceptable; otherwise the ECoG tracing was analyzed by a third person to seek consensus. Mice were defined as epileptic (PTE) or non-epileptic (No-SRS) based on the presence or absence of spontaneous seizures at either 3 or 5 months, or both (for cohort 1) or at 5 months (for cohort 2).

Cortical screw electrodes were similarly implanted in 5 naïve mice and in 5 sham mice (with craniectomy only) to monitor the effect of electrode implantation and surgery on the ECoG signal. These mice were used as control for the histological analysis.

### Brain tissue preparation for histological analysis

At the end of the experiment, TBI (n = 29) and control mice (n = 5 naïve and n = 5 sham) were deeply anesthetized (10% ketamine + 10% medetomidine + 80% saline; 10 ml/kg, i.p.) and perfused intracardially with ice-cold phosphate buffered saline (PBS, pH 7.4) followed by 4% paraformaldehyde (PAF) in PBS. Brains were removed from the skull and post-fixed for 90 min in 4% PAF in PBS at 4 °C, transferred to 20% sucrose in PBS for 24 h at 4 °C, then frozen in n-pentane for 3 min at − 50 °C, and stored at − 80 °C until assay. Coronal brain sections (30 μm) were cut on a cryostat from 0.98 mm to − 3.88 mm from bregma [43].

#### Nissl staining

Three antero-posterior levels (0.38 mm, − 1.70 mm and − 3.08 mm from bregma) were examined. Quantitative analysis was done in the striatum, thalamus (dorsal and ventral nuclei), septal pole of the hippocampus, and perilesional and entorhinal cortices. The ipsilateral cortex was analysed across two different antero-posterior levels (− 1.70 mm and − 3.08 mm from bregma), over a region 600 µm thick from the edge of the contusion. Striatum was quantified bilaterally, and thalamus, hippocampus and the entorhinal cortex were analysed only contralaterally, because of the brain cavity in the ipsilateral hemisphere of TBI mice. Two Nissl-stained slices per brain area 360 µm apart were matched for antero-posterior location in the various experimental group, and used for quantitative analysis.

Cell loss was measured as previously described [44, 45]. Briefly, images of the whole-brain coronal sections were captured at 20 × magnification using a Virtual Slide scanning microscopy system (Olympus, Germany) and digitized. In the hippocampus, neuronal cell loss was quantified by manually counting the number of Nissl-stained neurons in CA1 and CA3/CA4 pyramidal cell layers, and the hilar interneurons. For the remaining brain regions, images were processed using Fiji software [46]: an algorithm was created to segment and analyse every Nissl-stained cell over the entire manually selected ROI, as previously described [10, 28, 45]. Briefly, images were scaled into microns, and the background subtracted. Then an optimised threshold selected in a pilot study was applied across all the experimental groups to identify the Nissl-stained positive area, and the images were binarized. In order to unequivocally distinguish Nissl-stained neurons from glial cells and count only neuronal cells, we excluded from the counting those cells with an area below the cut-off of 25 µm^2^, using Fiji software [10, 28, 45]. Once segmented, all cells positive for Nissl were automatically quantified.

Data for each of the 2 slices/brain area/mouse were averaged, giving a single value for each brain area/mouse, and this value was used for statistical analysis.

### Randomization, blinding procedures and statistical analysis of data

A simple random allocation was applied to assign a subject to an experimental group (TBI or control). All evaluations were done blind to the sample identity.

Statistical analysis was done with GraphPad Prism 6 (GraphPad Software, USA) for Windows, using absolute values. The choice between parametric or non-parametric tests was based on passing the Shapiro–Wilk normality test, and data distribution was inspected by QQ plot. For each experiment, the figure legend reports the statistical analysis of data. We calculated the effect size (d) for all statistical comparisons, and we report only the exact d value associated to a large effect size (d ≥ 0.8) [47]. Data for TBI mice are presented as bargrams depicting the mean ± SEM and the single values (n = number of individual samples).

Differences between groups were reported as statistically significant for *p* < 0.05.

We assessed the performance of the measure(s) to distinguish PTE from No-SRS mice using non-parametric Receiver Operating Characteristics (ROC) curves: the area under the curve (AUC) was calculated and compared with chance (AUC = 0.5). The performance of the measure(s) was considered excellent for AUC values close to 1.

Mice omitted from data analysis are described in the figure legends, where appropriate.

## Results

### Detection of spontaneous seizures in TBI mice

Forty-five CD1 mice were exposed to TBI (cohort 1: n = 28; cohort 2: n = 17). Overall mortality was 35%: in cohort 1, 8 mice died within 1 month after TBI, and 2 mice died during the ECoG recording 3.5 months after TBI; in cohort 2, 2 mice died within 2 days after TBI, and 4 mice died during the ECoG recording 5 months after TBI. Thus, a total of 29 TBI mice (cohort 1, n = 18; cohort 2, n = 11) completed the longitudinal video-ECoG analysis for monitoring PTE.

#### Cohort 1

ECoG monitoring showed that 8 out of 18 TBI mice (45% of the injured animals) developed spontaneous recurrent motor seizures (SRS) 3.5 months after injury (pie chart in Fig. 2b). The incidence of PTE rose up to 58% by 5 months (11 out of 18 injured mice).

#### Cohort 2

On the basis of results of cohort 1, cohort 2 mice were ECoG recorded at 5 months post-injury: 6 out of 11 TBI mice developed PTE by 5 months (55% of injured animals), confirming the PTE incidence of cohort 1.

Overall, 17 out of 29 mice developed PTE (58% of injured animals), while the remaining 12 mice had no SRS throughout the recording time (pie charts in Fig. 2b). Naïve (n = 5) or sham (n = 5) mice did not experience seizures during the corresponding ECoG monitoring periods (Additional file 1: Figure S1).

ECoG seizures were observed in PTE mice either bilaterally in the injured and contralateral hemispheres (Fig. 2c), or unilaterally in the contralateral hemisphere only (Fig. 2d). The behavioral correlate of seizures included freezing, Straub tail and tail flicking, bilateral forepaw clonus, rearing and loss of posture. Tonic–clonic generalized seizures were also occasionally observed. PTE mice had one seizure every other day on average (number SRS/day, 3.5 months: 0.5 ± 0.2, n = 8; 5 months: 0.5 ± 0.1, n = 16): one mouse in cohort 1 had SRS at 3.5 months but not at 5 months (Fig. 2b). Average seizure duration rose significantly between 3.5 and 5 months post-TBI (sec, 3.5 months: 36.11 ± 5.76; 5 months: 53.64 ± 3.67; *p* < 0.05, d = 1.0; Fig. 2b).

On the basis of the presence of SRS at either 3 or 5 months, or both, mice were defined as epileptic (PTE, n = 17) or non-epileptic (No-SRS, n = 12). Body weight, neurological functions, MRI measures, and neuronal damage were retrospectively analysed and compared between the two groups.

### Evaluation of body weight

**Fig. 3:**
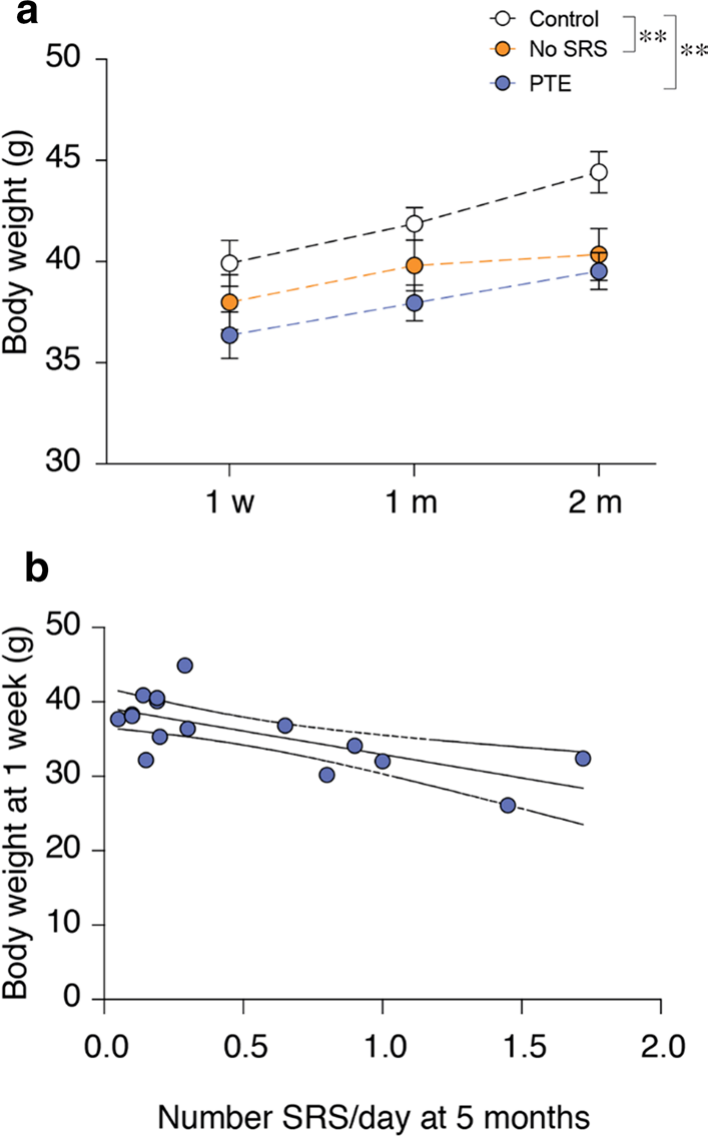
Body weight of mice with and without spontaneous seizures. **a** Longitudinal measures of body weight at 1 week, 1 month (controls: n = 18; PTE: n = 17; No-SRS: n = 12), and 2 months (controls: n = 8; PTE: n = 6; No-SRS: n = 5) after TBI. Data are mean ± SEM. **p < 0.01 versus controls by a mixed-effect model follow by Tukey’s post hoc multicomparison test. **b** Correlation between the body weight 1 week post-injury and the corresponding number of daily SRS at 5 months in PTE mice (n = 16; one PTE mouse in cohort 1 had SRS at 3.5 months but not at 5 months). Pearson correlation coefficient: r = − 0.69, *p* < 0.01

Body weight at 1 week, 1 month and 2 months after injury was significantly reduced in PTE (n = 17) and No-SRS (n = 12) groups to a similar extent as in controls (n = 18; *p* < 0.01, PTE: d = 1.1, No-SRS: d = 0.9) (Fig. 3a). Notably, in PTE mice, body weight 1 week post-TBI was negatively correlated with daily seizure frequency at 5 months (Pearson correlation coefficient: r = − 0.69, *p* < 0.01; Fig. 3b). No correlation was found between body weight and SRS frequency 1 and 2 months post-injury.

### Longitudinal evaluation of sensorimotor function

**Fig. 4:**
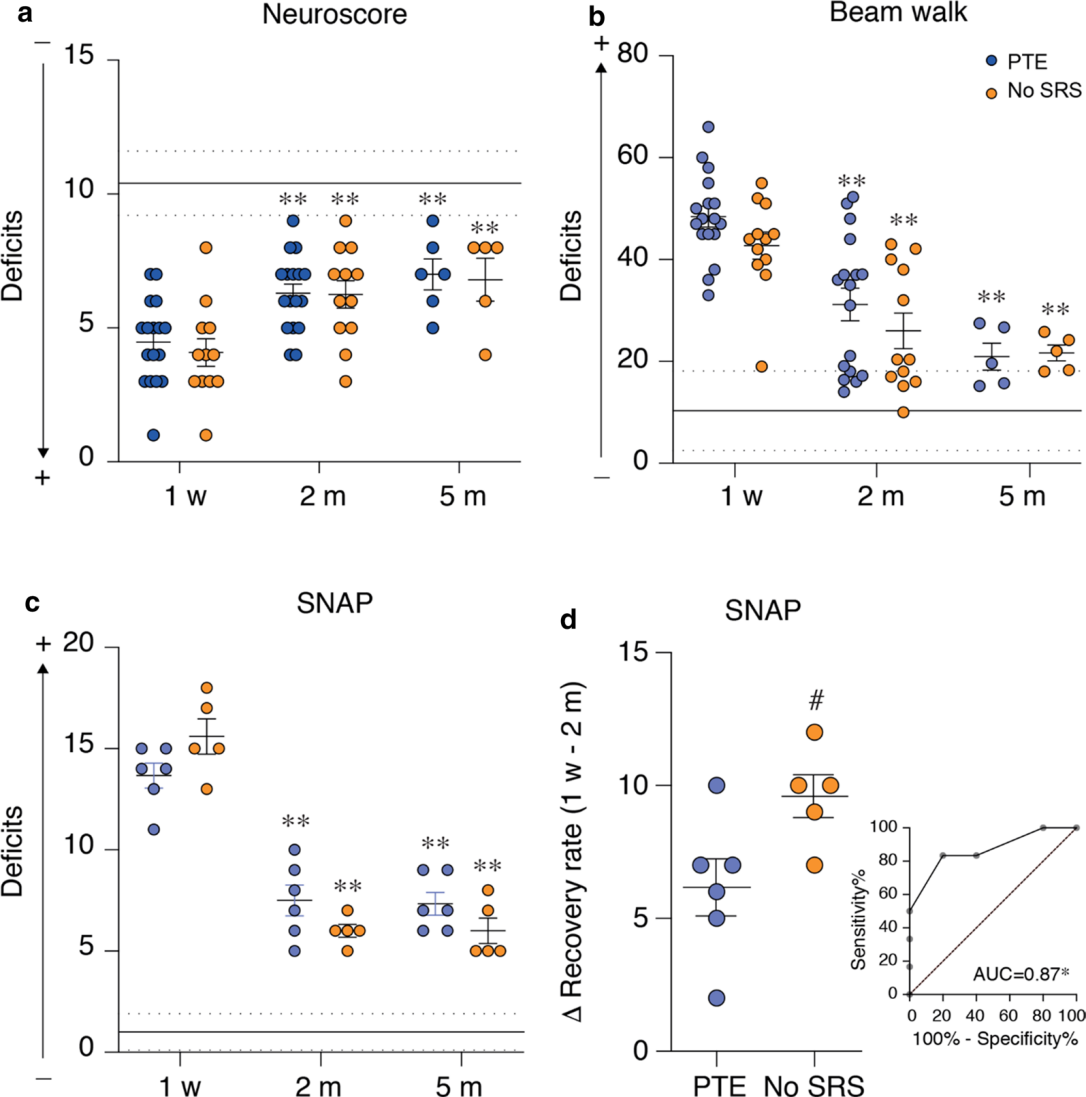
Longitudinal evaluation of sensorimotor function in mice with and without spontaneous seizures. **a**–**c** Sensorimotor deficits at 1 week, 2 months (controls: n = 18; PTE: n = 17; No-SRS: n = 12) and 5 months (controls: n = 8; PTE, n = 5–6; No-SRS, n = 5) post-TBI by neuroscore (**a**), beam walk (**b**; one PTE mouse at 5 months was identified as an outlier) and Simple Neuroassessment of Asymmetric impairment (SNAP) (**c**). The mean and SEM of control animals are shown by continuous and dotted black lines, respectively. Data are the mean ± SEM and the single values. **p < 0.01 versus 1 week in the same TBI group by a mixed-effect model followed by Tukey’s post hoc multicomparison test. **d** Recovery rate from 1 week to 2 months post-TBI evaluated by SNAP in PTE (n = 6) and No-SRS mice (n = 5). Data are the mean ± SEM and the single values. #*p* < 0.05, by two-tailed unpaired t-test. The insert shows the corresponding ROC analysis, *p* = 0.04

Sensorimotor function was examined by neuroscore and beam walk test 1 week and 2 months post-injury in all TBI mice, and at 5 months in cohort 2 only (*experimental plan in* Fig. 1). Initially, PTE (n = 17) and No-SRS mice (n = 12) had a similarly poor neuroscore of 3.9 and 4.4, respectively (*p* < 0.01 vs control mice, n = 18; PTE: d = 1.5, No-SRS: d = 1.8) with an improvement 2 months post-TBI (PTE: 6.0, d = 1.1; No-SRS: 6.2, d = 1.3; *p* < 0.01 vs 1 week) which was maintained at 5 months (PTE: n = 6, d = 1.3, No-SRS: n = 5, d = 1.3; control = 8; *p* < 0.01 vs 1 week; Fig. 4a). Beam walk was similarly impaired in both experimental groups, with initial scores of 48.4 in PTE (n = 17, *p* < 0.01, d = 1.8) and 44.1 in No-SRS mice (n = 12; *p* < 0.01, d = 1.8) compared to control mice (n = 18). Both scores similarly improved in TBI mice up to 31.2 (PTE) and 29.6 (No-SRS) by 2 months (*p* < 0.01 vs 1 week, PTE: d = 1.3, No-SRS: d = 0.9,), with no further improvement 5 months post-injury (controls: n = 8; *p* < 0.01 vs 1 week, PTE: n = 5, d = 1.9, No-SRS: n = 5, d = 1.9; Fig. 4b).

Cohort 2 mice were additionally exposed to the SNAP test. Similarly to the other behavioral tests, SNAP showed a deficit in TBI mice compared to controls (n = 8; *p* < 0.01, d = 1.5) at all time points, with no differences between PTE (n = 6) and No-SRS (n = 5) (Fig. 4c). PTE and No-SRS mice had initial scores of 13.7 and 15.6, respectively (*p* < 0.01 vs control, PTE: d = 1.9, No-SRS: n = 1.9), improving to 7.5 and 6 over the next 2–5 months (*p* < 0.01 vs 1 week, PTE: d = 1.8, No-SRS: d = 1.7; Fig. 4c). Interestingly, the recovery rate between 1 week and 2 months post-TBI (before the predicted PTE onset) was significantly lower in PTE mice (+ 45%) than No-SRS mice (+ 62%; *p* < 0.05, d = 1.2; Fig. 4d). The ROC curve comparing the recovery rate of PTE vs No-SRS mice gave an AUC = 0.87 (*p* = 0.04; 95% CI 0.6435–1.000; *insert in* Fig. 4c).

### Open field and cognitive deficits

Locomotor activity and anxiety-like behavior were evaluated in cohort 2 mice 1 month after TBI in the open field. PTE (n = 6) and No-SRS (n = 5) mice had locomotor activity similar to control mice (n = 8), measured as the total distance covered during the 5 min session (Additional file 1: Figure S2A). The time spent in the central zone of the arena, a measure of thigmotaxis and considered an index of anxiety-related behavior, was also similar to controls in both groups (Additional file 1: Figure S2B). Spatial memory 4 months post-TBI in the Y maze was similarly impaired in both PTE (n = 6, *p* < 0.01, d = 1.4) and No-SRS (n = 5, *p* < 0.05, d = 1.4) mice, as shown by the reduction of the time spent in the new arm of the maze compared to controls (n = 7; Additional file 1: Figure S2C).

### T2WI-based volumetric analysis

**Fig. 5:**
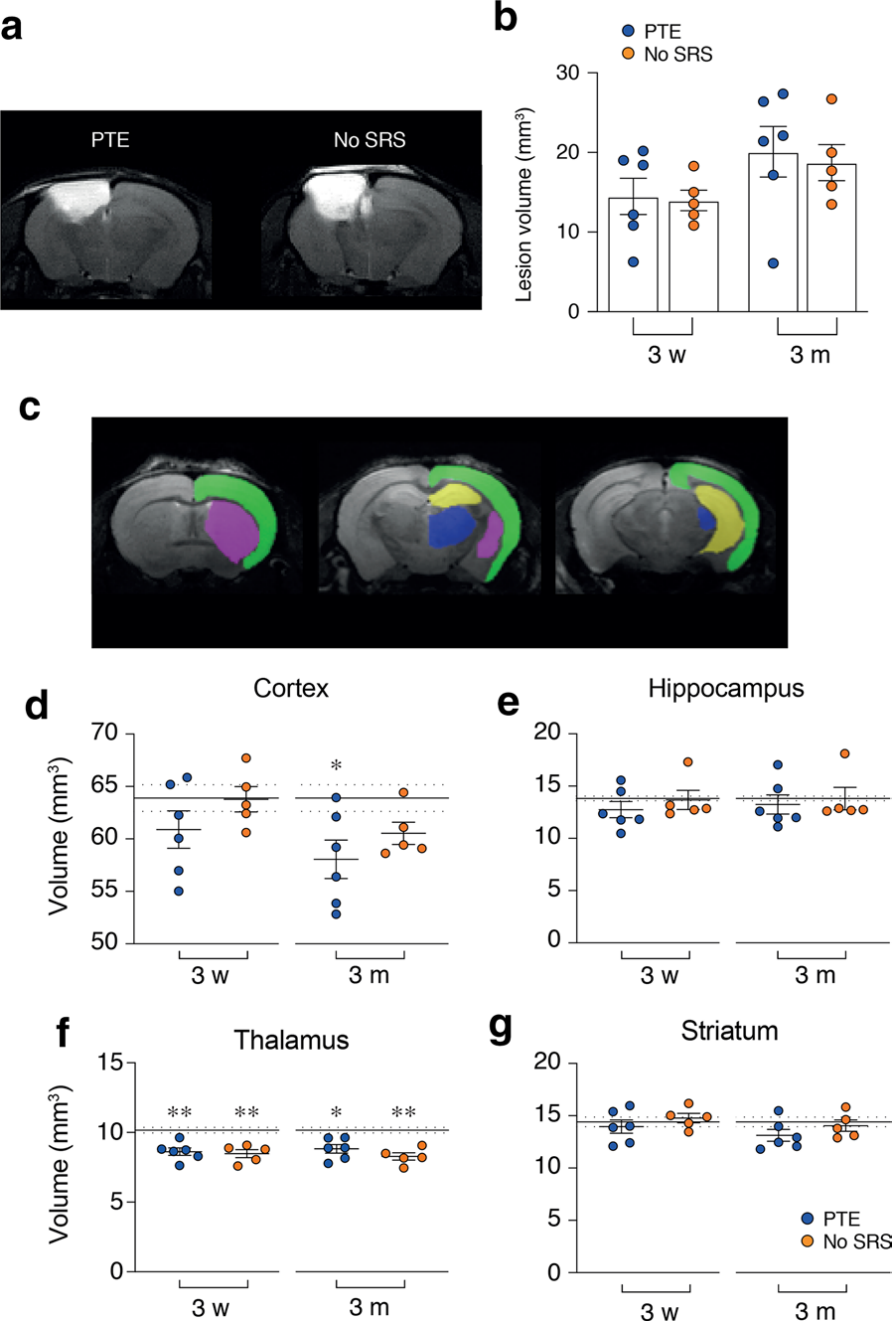
Brain volumetric analysis using T2WI in mice with and without spontaneous seizures. **a** T2WI showing brain coronal sections 3 months post-TBI in representative PTE (left) and No-SRS (right) mice. The white area corresponds to the lesion area. **b** Bargrams showing the longitudinal quantification of the lesion volume using T2WI images 3 weeks and 3 months post-TBI (PTE, n = 6; No-SRS, n = 5). Data are the mean ± SEM and the single values. **c** T2WI depicting representative brain coronal sections of a control mouse showing the ROIs used to quantify the volumes of selected brain regions, i.e. cortex (green), hippocampus (yellow), thalamus (blue), and striatum (pink). **d**–**g** Bargrams showing the volumetric analysis at 3 weeks and 3 months post-TBI (controls, n = 8; PTE, n = 6; No-SRS, n = 5). The mean and SEM of control animals are shown by continuous and dotted black lines, respectively. Statistical analyses were done using the corresponding contralateral values. Data are the mean ± SEM and the single values. *p < 0.05, **p < 0.01 versus corresponding contralateral control by Kruskall-Wallis

T2WI was done 3 weeks and 3 months post-injury in cohort 2 mice (*experimental plan* in Fig. 1). PTE and No-SRS mice had comparable lesion volumes at both times (mm^3^, 3 weeks, PTE: 14.5 ± 2.3, n = 6; No-SRS 14.0 ± 1.3, n = 5; 3 months, PTE: 20.1 ± 3.2, n = 6; No-SRS: 18.7 ± 2.3, n = 5; Fig. 5a, b). Contusion volume grew similarly over time in PTE (+ 38.7%) and No-SRS mice (+ 34.0%), approaching statistical significance (*p* = 0.06 in both experimental groups).

The volumes of selected forebrain regions were longitudinally measured in the contralateral hemisphere of injured mice (PTE, n = 6; No-SRS n = 5) and controls (n = 8). A reduction in the cortical volume was detected at 3 months in PTE mice only (*p* < 0.05, d = 1.2; Fig. 5d). Thalamus volume was similarly reduced both at 3 weeks and 3 months in PTE (*p* < 0.01, d = 1.5) and No-SRS mice (*p* < 0.01, d = 1.9) (Fig. 5f).

No volumetric changes were observed in the hippocampus and striatum of PTE and No-SRS mice compared to controls (Fig. 5e, g).

### Assessment of white matter integrity by DTI

**Fig. 6:**
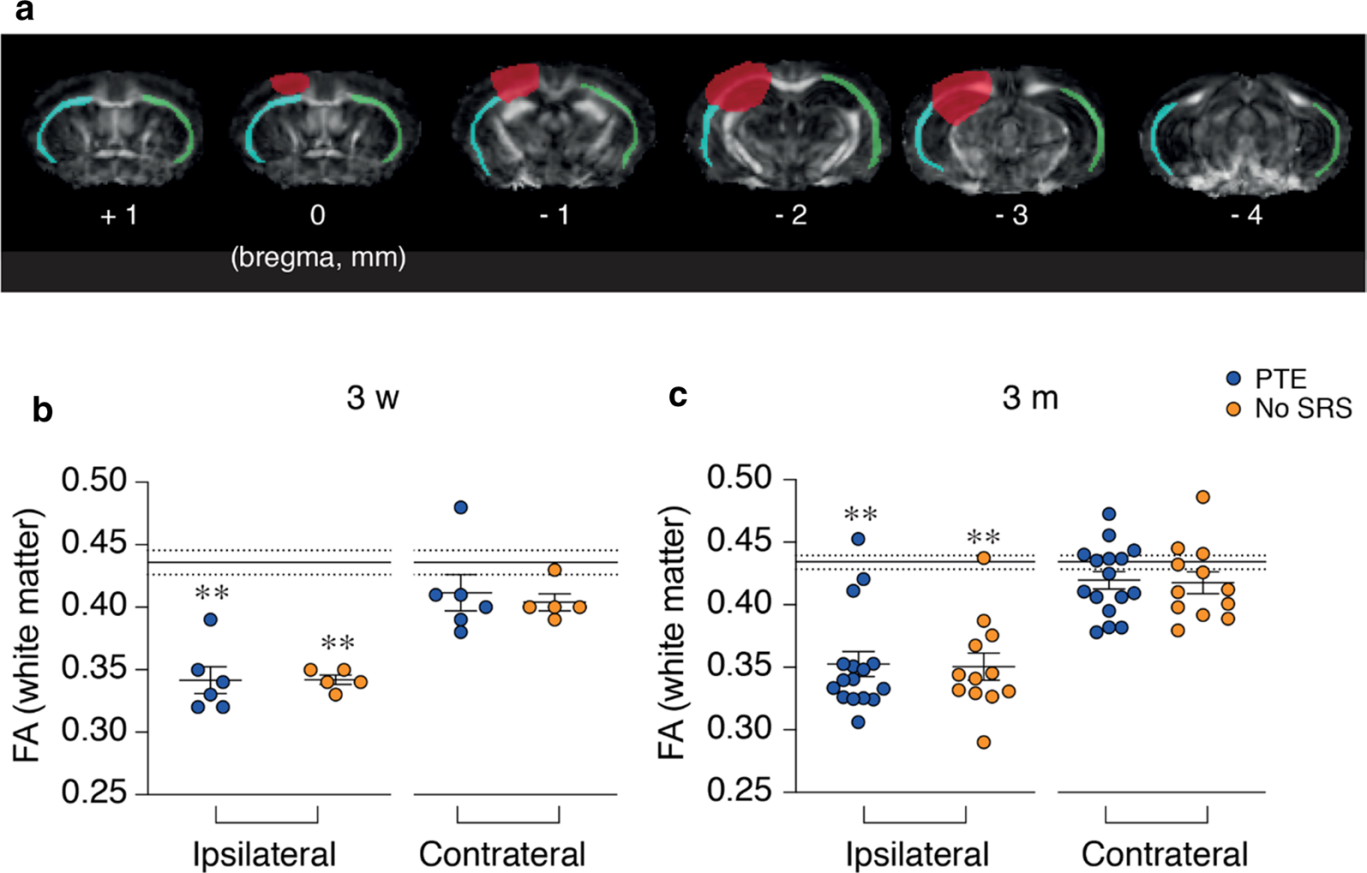
White matter integrity in mice with and without spontaneous seizures using DTI. **a** Representative images of brain coronal sections depicting the fractional anisotropy (FA) maps obtained by DTI 3 months after TBI at different antero-posterior levels in a representative PTE mouse. Contusion area (red); ipsilateral white matter (cyan line); contralateral white matter (green line). **b**, **c** Bargrams showing the quantification of FA 3 weeks (control: n = 6; PTE, n = 6; No-SRS, n = 5) and 3 months after TBI (controls: n = 17; PTE, n = 16, one control and one PTE mouse were discarded because of poor quality DTI images; No-SRS, n = 12) in the injured and contralateral hemisphere. The mean and SEM of control animals are shown by continuous and dotted black lines respectively, using the average of the two hemispheres. Statistical analyses were done using the corresponding ipsilateral or contralateral values. Data are the mean ± SEM and the single values. **p < 0.01 versus respective ipsilateral control by Kruskal–Wallis

White matter damage was evaluated by DTI 3 weeks post-injury in cohort 2 mice and at 3 months in both cohorts (*experimental plan in* Fig. 1). The analysis was done in the external capsule, which represents the largest preserved white matter tract in both ipsilateral and contralateral hemispheres (cyan and green lines, Fig. 6a). Compared to controls (n = 6–17), PTE and No-SRS mice had a significant lower FA values in the ipsilateral white matter at 3 weeks (− 22%, *p* < 0.01, d = 1.8 for both groups; Fig. 6b), persisting up to 3 months (− 20%, p < 0.01, PTE: d = 1.6, No-SRS: d = 1.7; Fig. 6c). No changes in FA were found in PTE and No-SRS animals in the contralateral white matter vs controls (Fig. 6b, c).

Changes in the other DTI metrics (AD, RD, MD) and reduction in white matter volumes were detected over time in PTE and No-SRS mice compared to control animals; however, none of these parameters distinguished PTE from No-SRS mice (Additional file 1: Figures S3 and S4).

### Evaluation of neuronal cell loss

Quantitative analysis of cell loss was done in matched Nissl-stained brain regions of control (naïve, n = 5; sham, n = 5), PTE (n = 11) and No-SRS (n = 9) mice taken randomly from cohorts 1 and 2 at the end of ECoG monitoring. Analysis was done in the parieto-temporal cortex and striatum of both hemispheres. Because of extensive tissue loss induced by TBI in the ipsilateral hemisphere, the hippocampus, thalamus, and entorhinal cortex were quantified only in the contralateral hemisphere.

**Fig. 7:**
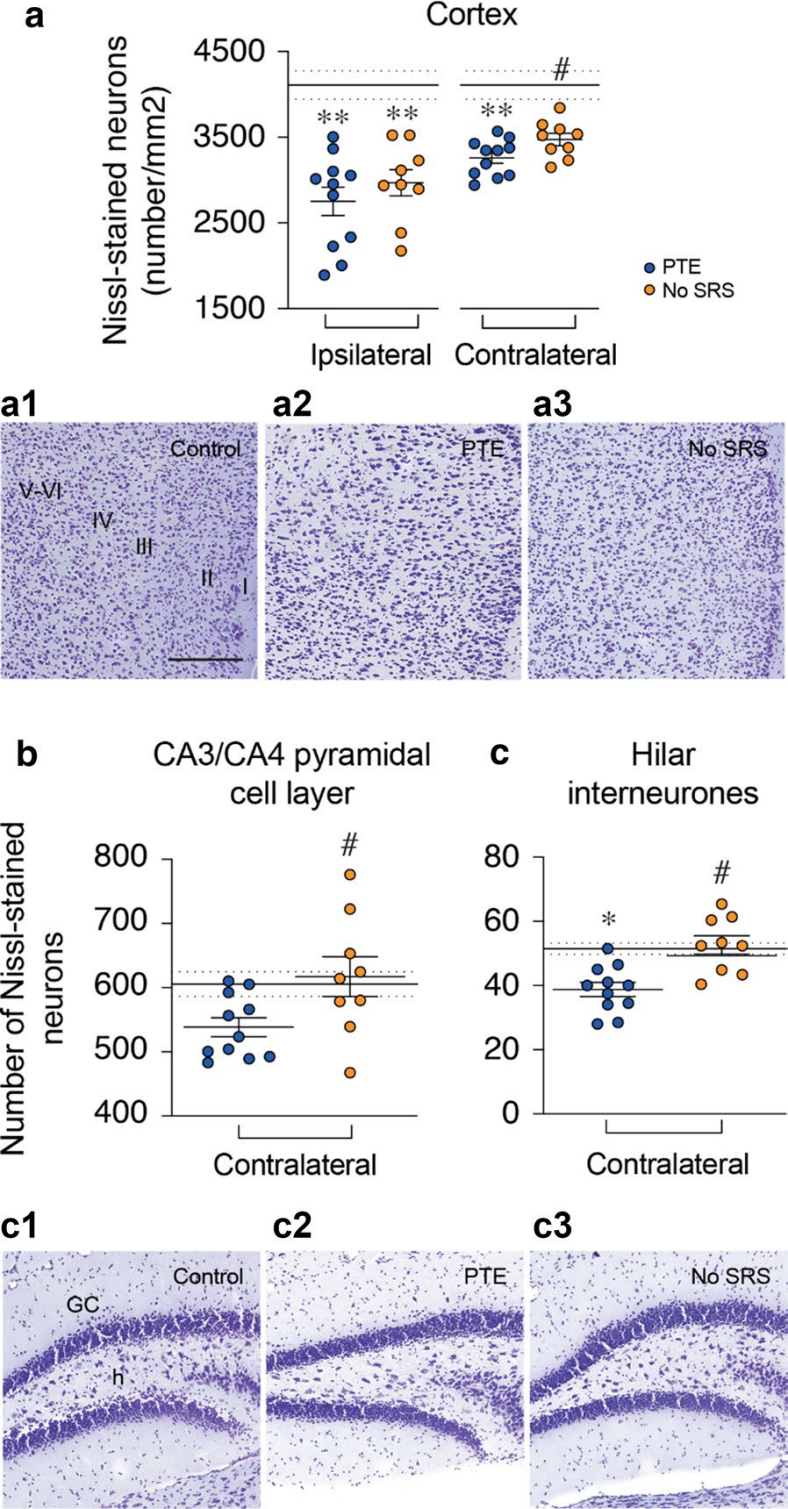
Neurodegeneration in the cortex, CA3/4 pyramidal cell layer and hilus of the dentate gyrus of mice with and without spontaneous seizures. **a**–**c** Quantitative analysis of neuronal cell loss in the cortex (**a**), CA3/4 pyramidal cell layer (**b**), and hilar interneurons (**c**) in control mice (n = 10) and in PTE (n = 11) and No-SRS (n = 9) mice. The mean and SEM of control animals are shown by continuous and dotted black lines, respectively. Data are the mean ± SEM and the single values. *p < 0.05, **p < 0.01 vs corresponding hemisphere in controls by one way ANOVA followed by Tukey’s post hoc multicomparison test; **a** #*p* < 0.05 vs PTE of the same hemisphere by two tailed unpaired t-test; **b**, **c** #*p* < 0.05 vs PTE by one-way ANOVA followed by Tukey’s post hoc multicomparison test. **a1**–**a3**, **c1**–**c3** Photomicrographs depicting representative Nissl-stained contralateral cortices (**a1**–**a3**), and hilar interneurons (**c1**–**c3**) in the experimental groups. Scale bar: 150 µm

Neuronal cell loss occurred bilaterally in the parieto-temporal cortex of PTE mice, extending − 1.7 to − 3.08 mm from bregma compared to control mice (perilesional: -34%, *p* < 0.01, d = 1.6; contralateral: − 28%, *p* < 0.01, d = 1.5) (Fig. 7a). No-SRS mice had similar reductions in the number of neurons in the ipsilateral cortex (− 23%, *p* < 0.01, d = 1.5), while contralateral cortical neurodegeneration was significantly higer in PTE mice (-22% in PTE mice vs − 15% in No-SRS mice, *p* < 0.05, d = 1.1) (Fig. 7a, a1–a3)

In the contralateral hippocampus, PTE mice had CA1 pyramidal neuron loss (− 16%, *p* < 0.05, d = 1.1; Additional file 1: Figure S5A) and fewer hilar interneurons (− 25%, d=1.3, *p* < 0.01; Fig. 7b) compared to control mice. CA3/4 pyramidal cells were also reduced, although not significantly (− 13%, *p* = 0.08, d = 1.0; Fig. 7b). No-SRS mice had no neurodegeneration in the same brain areas (Fig. 7b, c, c1–c3; Additional file 1: Figure S5A).

The striatum ipsilateral to the injured site showed similar neurodegeneration in PTE (− 17%, *p* < 0.05, d = 1.2) and No-SRS mice (− 16%, *p* < 0.05, d = 1.4) compared to control mice (Additional file 1: Figure S5C), while the contralateral striatum displayed no cell loss in both experimental groups (Additional file 1: Figure S5B).

The contralateral thalamus showed similar neurodegeneration in PTE (− 20%, n = 11; *p* < 0.01, d = 1.5; Additional file 1: Figure S5C) and No-SRS mice (− 25%, n = 9, *p* < 0.01, d = 1.5; Additional file 1: Figure S5C) compared to controls (n = 10). No neuronal cell loss was found in the contralateral entorhinal cortex of injured mice in either group (Additional file 1: Figure S5D).

## Discussion

We report new evidence from in-depth characterisation of a mouse model of PTE based on longitudinal ECoG recording, behavioural tests of neurological dysfunctions and structural MRI analysis, together with post-mortem histopathological assessment. We used CD1 mice since this strain has higher susceptibility to PTE development compared to C57BL6 mice [15–19]. In accordance with high PTE incidence in CD1 mice exposed to severe CCI [19], we found that 58% of TBI-exposed mice develop epilepsy by 5 months with frequent seizures. This model is therefore highly valuable for determining biomarkers of epileptogenesis and for testing disease-modifying interventions. Available clinical indicators identify patients at high risk for PTE with a prediction rate of 20–30% [2]; biomarker discovery in this enriched PTE model may help to increase the rate of epilepsy prediction in the severe TBI patient population.

In line with previous studies, we found that TBI mice presented early significant impairment in sensorimotor functions, with partial recovery at chronic stages [10, 27–29]. When searching for potential biomarkers, we found similar sensorimotor deficits such as composite neuroscore and beam walk in all TBI mice regardless of PTE development, as also described in the rat lateral FPI model [48] and compatible with clinical measures of neurological deficits [49]. Notably, the rate of functional recovery measured by the SNAP test was significantly lower in PTE mice than No-SRS mice. The ROC curve showed that the SNAP recovery rate allowed accurate distinction between PTE and No-SRS mice. Importantly, this difference is captured before the predicted time of PTE onset in this model [19], indicating that the rate of neurological recovery in TBI patients may provide a sensitive measure for predicting PTE development. Since SNAP test was performed only in one cohort of mice, further validation is needed to confirm its prognostic value for PTE.

Additionally, we found that loss of body weight early after injury in PTE mice correlated with their seizure frequency in the chronic epilepsy phase, thus providing a potential biomarker for disease severity. Rats developing PTE after FPI showed greater body weight loss (up to 1 week post-injury) compared to TBI rats not developing epilepsy [48]. The mechanisms underlying the association between early loss in body weight and PTE are not known. Whether the concomitant early epileptiform ECoG alterations (up to 1 week) in TBI mice prone to epilepsy reduces feeding, thus causing weight loss, or whether other brain or systemic factors are involved, is worth investigating since it may give clues to brain activity or functional changes predictive of epilepsy development.

Neuronal cell loss in forebrain areas of the injured hemisphere, as well as contusion volume and white matter damage, shown by MRI analysis, were similar in PTE and No-SRS mice. However, quantification of neuronal cell loss in the hemisphere contralateral to the biomechanical impact showed cortical and hippocampal neurodegeneration in PTE mice, but not in No-SRS mice. This new evidence highlights the need for carefully assessing neurodegeneration also outside the areas of primary injury, and beyond the perilesional tissue. In particular, neurodegeneration in the contralateral hippocampus of PTE mice resembled hippocampal damage in experimental and human temporal lobe epilepsy, with loss of CA1 and CA3/4 pyramidal cells and hilar interneurons. In fact, 35–56% of PTE patients have seizures arising from the mesial temporal region [50, 51]. The loss of hilar inhibitory interneurons may play a key role in PTE development since transplantation of GABAergic progenitors in CD1 mice exposed to CCI migrate mostly into the hilus and prevent PTE [52]. Similarly, interneuron transplantation in the hippocampus of mice with temporal lobe epilepsy dramatically reduced seizure frequency [53]. Whether neuronal cell loss in the contralateral hemisphere is induced by the spread of subclinical hyperexcitability from the injured site then contributing to seizures or is a consequence of seizures remains to be established; however, in some cases we detected seizures occurring only in the contralateral cortex in PTE mice.

The neurodegeneration in the contralateral hemisphere of PTE mice prompts further studies on the mechanisms underpinning this increased vulnerability to cell death. Imbalances between excitatory and inhibitor neurotransmission, together with neuroinflammation, oxidative stress and blood–brain barrier dysfunction are pathologic processes likely involved in neuronal injury. Longitudinal molecular and functional brain MRI analyses mirroring these events (i.e., ^1^H-MRS, CEST- and gadolinium-based imaging) should help to elucidate the neuropathological sequelae associated with PTE using non-invasive approaches, thus providing potential biomarkers. Additionally, the specific vulnerability of hilar interneurons in PTE mice warrants a detailed histological characterisation of the dentate gyrus and hilar region during post-traumatic epileptogenesis (e.g., mossy fiber sprouting, neurogenesis, phenotypic identification of hilar interneurons) to gain insights into mechanisms of aberrant excitability leading to seizure generation.

Volumetric quantification of tissue damage in selected forebrain areas in the contralateral side by T2WI analysis did not show significant differences between PTE and No-SRS mice, similarly to the lack of distinction of volumetric changes in the injured hemisphere after FPI [54]. Interestingly, hippocampal surface shape analysis 1 week after FPI using large-deformation high-dimensional mapping identified morphometric changes compared to baseline (before trauma) that were different in PTE and non PTE rats [54]. These findings suggest that subtle structural abnormalities rather than major structural changes are likely to be associated with post-traumatic epileptogenesis.

The biomechanical impact of TBI leads to diffuse axonal injury across the brain. Accordingly, white matter injury has been detected in patients, persisting for several years after TBI, as assessed by either histopathology or DTI [55, 56]. We found that FA was similarly reduced in both PTE and No-SRS mice compared to controls up to 3 months post-TBI, which corresponds to the time of disease onset in the model [19]. In human TBI, FA is significantly lower in patients with chronic epilepsy than TBI patients without epilepsy [57]. This clinical finding suggests that a longitudinal high-resolution DTI analysis, including the chronic phase of the disease (i.e., up to 5 months post-TBI), is warranted in our model to possibly identify DTI measures associated with acquired PTE. In particular, neurite orientation dispersion and density imaging (NODDI), an MRI measure more sensitive than classical DTI parameters [58], may allow the identification of microstructural changes of dendrites and axons [59], which could inform on PTE development.

In conclusion, our study provides a comprehensive characterisation of a model of severe TBI evolving to PTE in CD1 mice. We identified early behavioral measures that distinguish mice developing epilepsy or not, and predict seizure frequency in the PTE group, therefore serving as potential prognostic biomarkers of epileptogenesis. Histological examination of neuronal damage highlighted notable differences between mice with or without PTE in the hemisphere contralateral to the primary injury, that cannot be detected by MRI. This suggests that advanced MRI image processing methods, permitting the prediction of histopathological features in vivo with high accuracy and previously used in epilepsy patients [60–62], should be considered to define the pivotal structures within the epileptogenic circuitry in TBI-exposed people.

Since this PTE model recapitulates key features of contusive TBI in patients, it could serve as a clinically relevant model for advancing both the discovery and the validation of biomarkers of epileptogenesis, and for developing novel preventive or disease-modifying pharmacological interventions.

## Supplementary Information

**Additional file 1.** Figure S1: Representative ECoG traces of control mice; Figure S2: Locomotor activity, anxiety-like behavior and cognitive deficits in mice with and without spontaneous seizures; Figure S3: AD, RD and MD in mice with and without spontaneous seizures using DTI; Figure S4: Quantification of while matter volume in mice with and without spontaneous seizures using DTI; Figure S5: Neurodegeneration in the CA1 pyramidal layer, striatum, thalamus and entorhinal cortex of mice with and without spontaneous.

## Data Availability

Data from this study are available on reasonable request to the corresponding author.

## Abbreviations

AUC: Area under the curve
CCI: Controlled cortical impact
DTI: Diffusion tensor imaging
ECoG: Electrocorticography
MRI: Magnetic resonance imaging
PTE: Post-traumatic epilepsy
ROC: Receiver operating characteristics
SNAP: Simple neuroassessment of asymmetric impairment
SRS: Spontaneous recurrent seizures
T2WI: T2-weighted image
TBI: Traumatic brain injury
