# Supplementary material for: In-depth characterization of a mouse model of post-traumatic epilepsy for biomarker and drug discovery
Source: Acta Neuropathol Commun. 2021 Apr 26;9:76. doi: 10.1186/s40478-021-01165-y (PMC8073903; doi:10.1186/s40478-021-01165-y)
Supplement: Supplementary file 1 — Additional file 1. Figure S1: Representative ECoG traces of control mice; Figure S2: Locomotor activity, anxiety-like behavior and cognitive deficits in mice with and without spontaneous seizures; Figure S3: AD, RD and MD in mice with and without spontaneous seizures using DTI; Figure S4: Quantification of while matter volume in mice with and without spontaneous seizures using DTI; Figure S5: Neurodegeneration in the CA1 pyramidal layer, striatum, thalamus and entorhinal cortex of mice with and without spontaneous. [file 40478_2021_1165_MOESM1_ESM.pdf]

Supplementary Material

In-depth characterization of a mouse model of post-traumatic epilepsy for biomarker and drug discovery

Rossella Di Sapia[1*], Federico Moro[1*], Marica Montanarella[1], Valentina Iori[1], Edoardo Micotti[1], Daniele Tolomeo[1], Kevin K.W. Wang[2], Annamaria Vezzani[1], Teresa Ravizza[1] and Elisa R. Zanier[1]

Department of Neuroscience[1], Istituto di Ricerche Farmacologiche Mario Negri IRCCS, Milano, Italy; [2]Program for Neurotrauma, Neuroproteomics & Biomarkers Research, Departments of Emergency Medicine, Psychiatry, Neuroscience and Chemistry, University of Florida, Gainesville, FL, USA

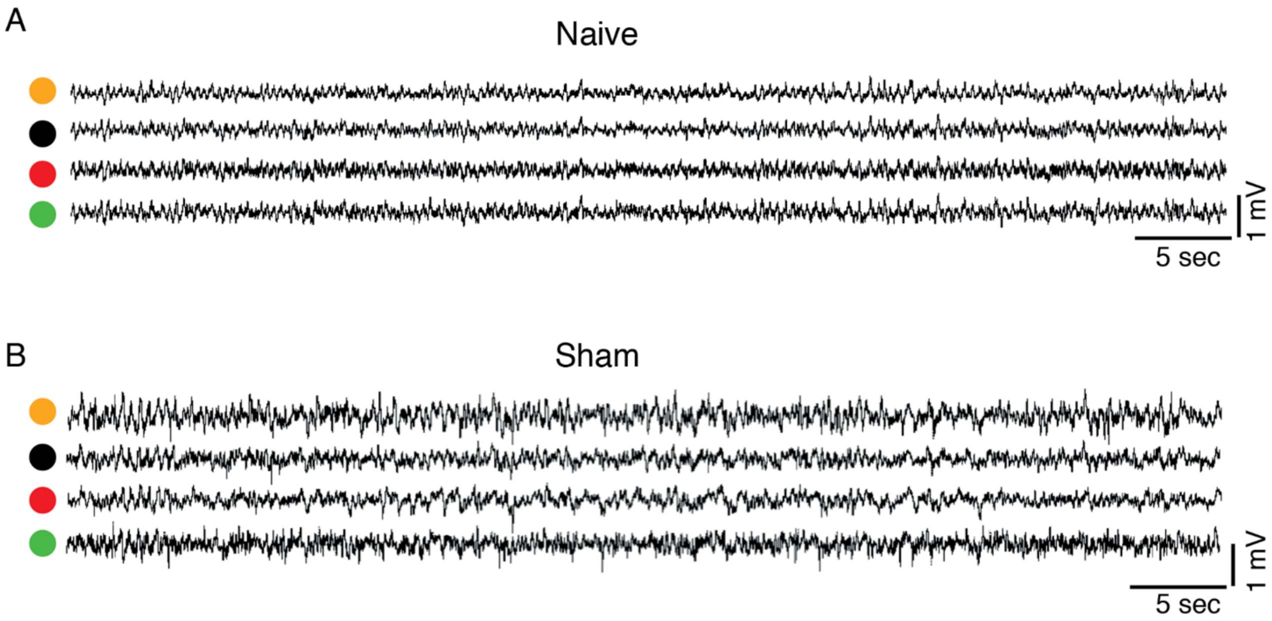

Suppl. Figure 1. *Representative ECoG traces of control mice.*

Representative ECoG tracings of naïve (electrode implantation only; A, n=5) and sham control mice (craniectomy and electrode implantation; B, n=5) 5 months after TBI. The color code at the beginning of the traces identifies the electrode used for the recording (see Figure 2B for details).

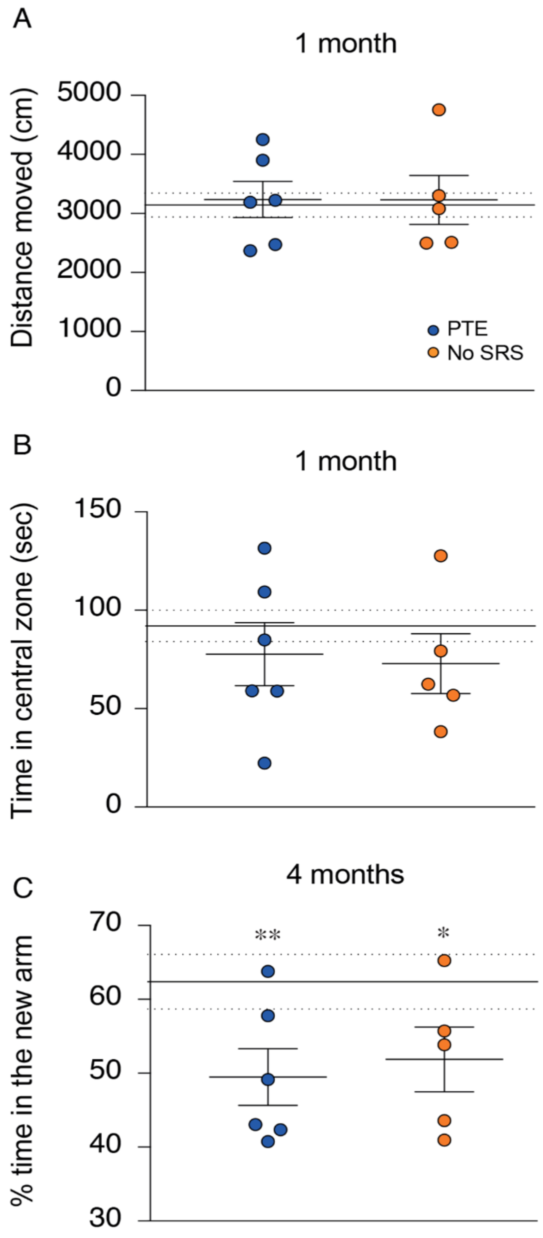

Suppl. Figure 2. *Locomotor activity, anxiety-like behavior and cognitive deficits in mice with and without spontaneous seizures.*

Panels A-B. Graphs show the total distance covered (A) and the time spent in the central zone (B) measured 1 month post-TBI in PTE (n=6) and No-SRS mice (n=5) and controls (n=8) in the open field.

Panel C. Graphs showing the percentage of time spent exploring the new arm of the Y maze during the retrieval phase (trial 2) 4 months post-TBI in PTE (n=6), and No-SRS (n=5) mice, and controls

(n=7, one control was identified as an outlier). *p<0.05, **p<0.01 vs controls by one-way ANOVA followed by Tukey's *post hoc* multicomparison test.

In panels A-C, the mean and SEM of control animals are shown by continuous and dotted black lines, respectively. Data are the mean ± SEM and the single values.

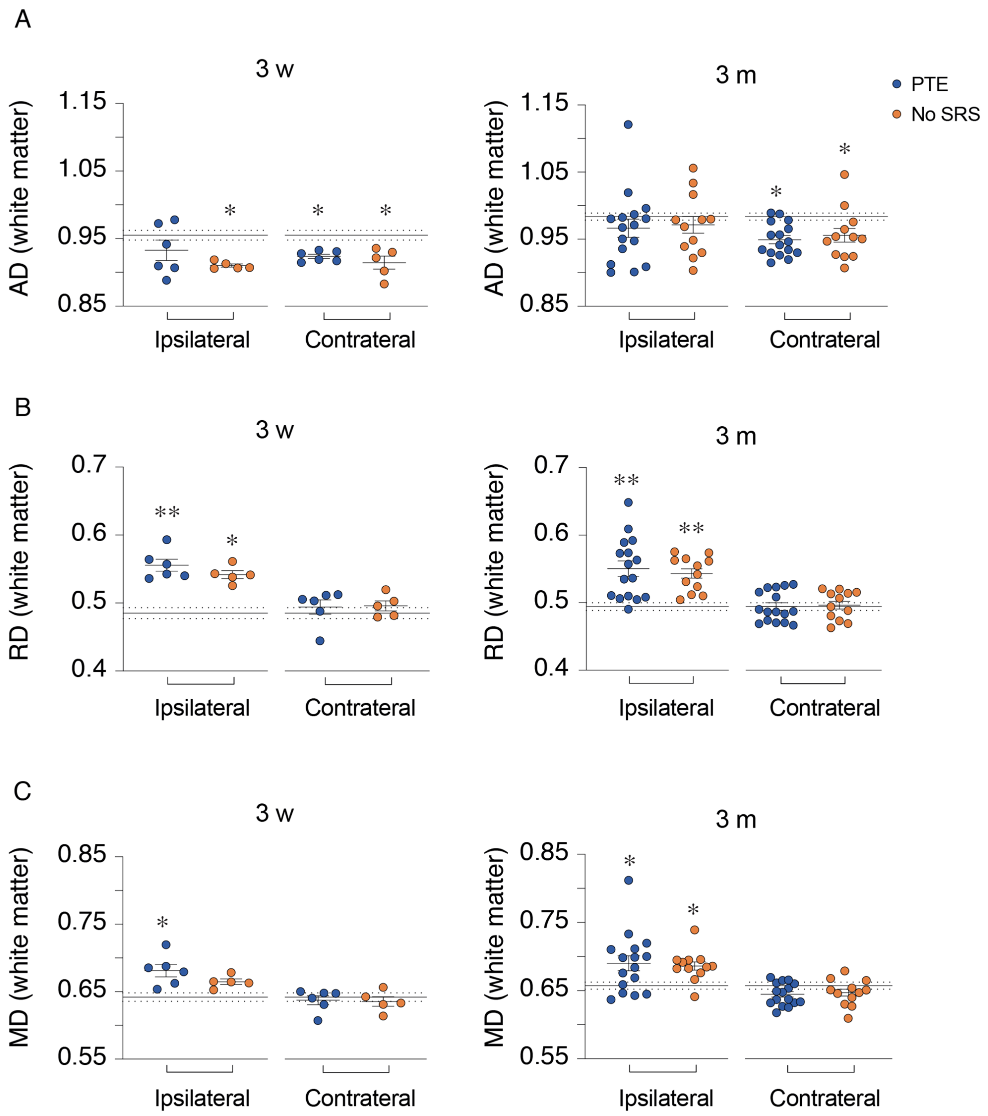

Suppl. Figure 3. *AD, RD and MD in mice with and without spontaneous seizures using DTI.*

Panels A-C. Bargrams show the quantification of AD (A), RD (B) and MD (C) at 3 weeks (control: n=6; PTE, n=6; No- SRS, n=5) and 3 months after TBI (controls: n=17; PTE, n=16, one control and one PTE mouse were discarded because of poor quality images; No-SRS, n=12) in the injured and contralateral hemisphere. The mean and SEM of control animals are shown by continuous and dotted black lines, respectively, using the average of the two hemispheres. Statistical analysis was done using the corresponding ipsilateral or contralateral values. Data are the mean ± SEM and the single values. *p<0.05, **p<0.01 *vs* respective ipsilateral or contralateral control by Kruskall-Wallis.

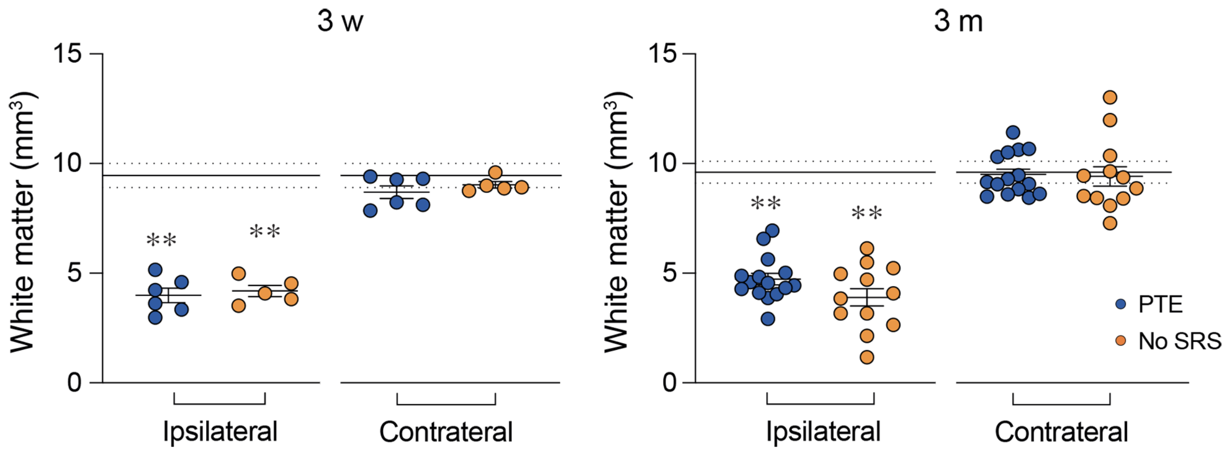

Suppl. Figure 4. *Quantification of while matter volume in mice with and without spontaneous seizures using DTI.*

Bargrams show the quantification of white matter volumes at 3 weeks (control: n=6; PTE, n=6; No-SRS, n=5) and 3 months after TBI (controls: n=17; PTE, n=15, one control and one PTE mouse were discarded because of poor quality images, one additional PTE mouse was identified as an outlier; No-SRS, n=12) in the injured and contralateral hemisphere. The mean and SEM of control animals are shown by continuous and dotted black lines, respectively, using the average of the two hemispheres. Statistical analysis was done using the corresponding ipsilateral or contralateral values. Data are the mean ± SEM and the single values. **p<0.01 *vs* respective ipsilateral control by Kruskall-Wallis.

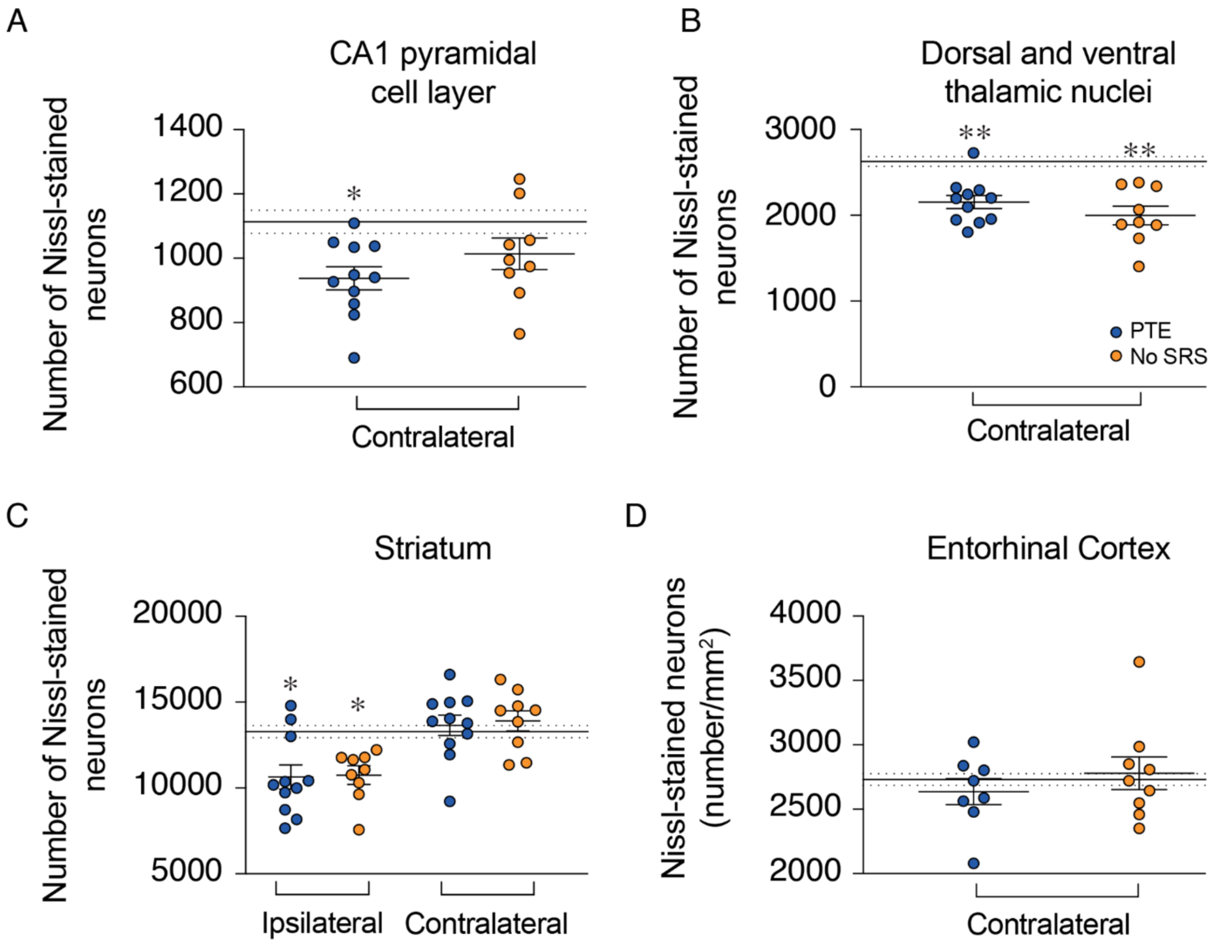

Suppl. Figure 5. *Neurodegeneration in the CA1 pyramidal layer, striatum, thalamus and entorhinal cortex of mice with and without spontaneous.*

Panels A-D. Quantitative analysis of neurodegeneration in the CA1 pyramidal cell layer (A), thalamus (B), striatum (C), and entorhinal cortex (D) of sham mice (n=10) and PTE mice (n=8-11), and No-SRS (n=9) mice. Entorhinal cortex: one PTE mouse was identified as an outlier, and two PTE mice were discarded because of poor-quality brain slices. The mean and SEM of control animals are shown by continuous and dotted black lines, respectively. Panel C: statistical analyses were done using the corresponding ipsilateral or contralateral values. Data are mean ± SEM and the single values. *$p<0.05$, **$p<0.01$ vs corresponding hemisphere in controls by one-way ANOVA followed by Tukey's *post hoc* multicomparison test.
